# Morpholin-4-ium morpholine-4-carbo­dithio­ate

**DOI:** 10.1107/S1600536811026286

**Published:** 2011-07-13

**Authors:** Ana C. Mafud, Edgar A. Sanches, Maria Teresa Gambardella

**Affiliations:** a Instituto de Química de São Carlos, Universidade de São Paulo, Av. Trabalhador Sãocarlense, 400, Caixa Postal 780, 13560-970, São Carlos SP, Brazil

## Abstract

The title compound, C_4_H_10_NO^+^·C_5_H_8_NOS_2_^−^, is built up of a morpholinium cation and a dithio­carbamate anion. In the crystal, two structurally independent formula units are linked *via* N—H⋯S hydrogen bonds, forming an inversion dimer, with graph-set motif *R*_4_^4^(12).

## Related literature

For the crystal structures of similar compounds, see: Wahlberg (1979 ▶, 1980 ▶, 1981 ▶); Mafud & Gambardella (2011*a* ▶,*b* ▶). For graph-set analysis, see: Bernstein *et al.* (1995 ▶). For puckering parameters, see: Cremer & Pople (1975 ▶).
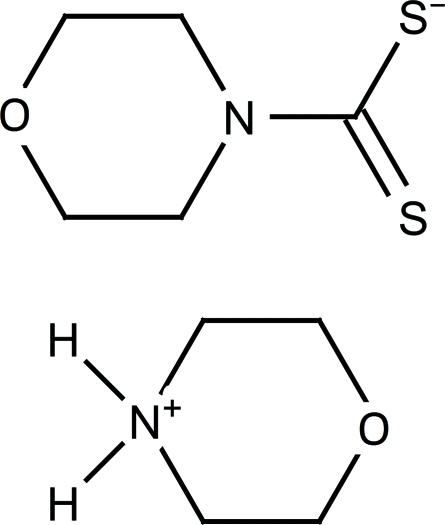


## Experimental

### 

#### Crystal data

C_4_H_10_NO^+^·C_5_H_8_NOS_2_^−^
*M* *_r_* = 250.37
Monoclinic, 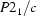

*a* = 7.938 (5) Å
*b* = 18.3232 (15) Å
*c* = 8.8260 (5) Å
β = 110.021 (5)°
*V* = 1206.2 (8) Å^3^
*Z* = 4
Mo *K*α radiation
μ = 0.43 mm^−1^
*T* = 290 K
0.3 × 0.15 × 0.15 mm

#### Data collection

Enraf–Nonius TurboCAD-4 diffractometer
Absorption correction: ψ scan (North *et al.*, 1968 ▶) *T*_min_ = 0.795, *T*_max_ = 0.902
3705 measured reflections
3487 independent reflections
2021 reflections with *I* > 2σ(*I*)
*R*_int_ = 0.041
3 standard reflections every 120 min  intensity decay: 5%

#### Refinement

*R*[*F* ^2^ > 2σ(*F* ^2^)] = 0.050
*wR*(*F* ^2^) = 0.145
*S* = 1.00
3487 reflections
190 parameters
All H-atom parameters refined
Δρ_max_ = 0.56 e Å^−3^
Δρ_min_ = −0.39 e Å^−3^

### 

Data collection: *CAD-4 EXPRESS* (Enraf–Nonius, 1989) ▶; cell refinement: *CAD-4 EXPRESS* ▶; data reduction: *XCAD4* (Harms & Wocadlo, 1995 ▶); program(s) used to solve structure: *SIR92* (Altomare *et al.*, 1994 ▶); program(s) used to refine structure: *SHELXL97* (Sheldrick, 2008 ▶); molecular graphics: *ORTEP-3 for Windows* (Farrugia, 1997 ▶) and *Mercury* (Macrae *et al.*, 2006 ▶); software used to prepare material for publication: *WinGX* (Farrugia, 1999 ▶), *PLATON* (Spek, 2009 ▶) and *publCIF* (Westrip, 2010 ▶).

## Supplementary Material

Crystal structure: contains datablock(s) I, global. DOI: 10.1107/S1600536811026286/su2285sup1.cif

Structure factors: contains datablock(s) I. DOI: 10.1107/S1600536811026286/su2285Isup2.hkl

Supplementary material file. DOI: 10.1107/S1600536811026286/su2285Isup3.cml

Additional supplementary materials:  crystallographic information; 3D view; checkCIF report

## Figures and Tables

**Table 1:** Hydrogen-bond geometry (Å, °)

*D*—H⋯*A*	*D*—H	H⋯*A*	*D*⋯*A*	*D*—H⋯*A*
N2—H1*N*⋯S1	0.86 (4)	2.47 (4)	3.284 (3)	158 (3)
N2—H2*N*⋯S1^i^	0.91 (4)	2.75 (4)	3.453 (2)	135 (3)
N2—H2*N*⋯S2^i^	0.91 (4)	2.39 (3)	3.221 (2)	151 (3)

Symmetry code: (i) 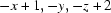.

**Fig. 1.**
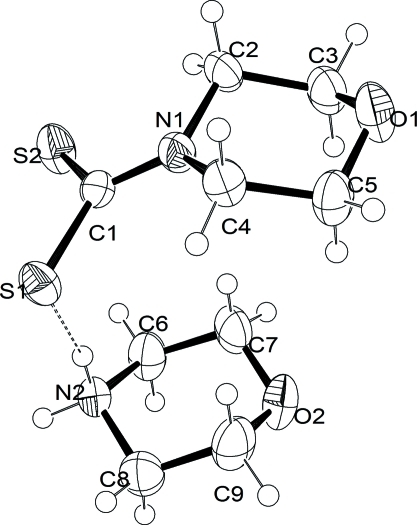
Perspective view of the molecular structure of the title salt, with numering scheme and displacement ellipsoids drawn at the 50% probability level.

**Fig. 2.**
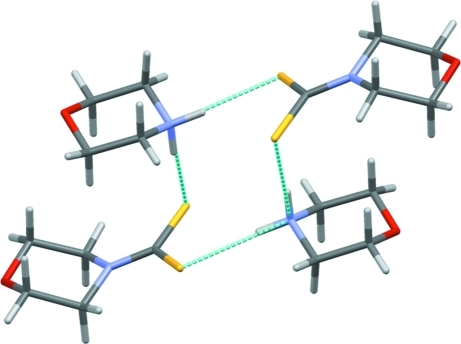
Perspective view of the N-H···S hydrogen bonded (dashed cyan lines) dimer in the title salt, with graph-set R44(12).

## supplementary crystallographic information

### Comment

The first thiocarbamic acid-ammonium salt, pyrrolidinedithiocarbamic acid-pyrrolidineammonium salt, was reported on previously by (Wahlberg, 1979; 1980; 1981). Our group have recently described the synthesis and crystal structures of ammonium piperidine-1-carbodithioate and sodium piperidine-1-carbodithioate dihydrate (Mafud & Gambardella, 2011*a*,*b*). Continuing our research on this subject, we report herein on the synthesis and crystal structure of the title salt, 1-Morpholinedithiocarbamic Acid-morpholineammonium Salt.

In the molecular structure of the title compound (Fig. 1) there is an intramolecular hydrogen bond involving the cation, via the nitrogen atom from amine group, and the anion, via the sulfur atom of dithiocarbamate (Table 1). The six membered rings have chair conformations, with puckering parameters are Q=0.554 (3) Å, θ = 177.4 (3)°, φ2 = 168 (6)° for the anion and Q = 0.566 (3) Å, θ = 1.4 (4)°, φ2 = 60 (14)° for the cation (Cremer & Pople, 1975).

In the crystal two structurally independent formula units are linked via N—H···S hydrogen bonds (Fig. 2, Table 1), to form a dimer arrangement centered about an inversion center, with graph-set R^4^_4_(12) [Bernstein *et al.*, 1995].

### Experimental

The RNH_2_^+^ salt of the morpholinedithiocarbamate was prepared by slow addition of 0.1 mol of CS_2_ to a cold solution (ice bath) containing 0.2 mol of the morpholien amine dissolved in 30 ml of ethanol-water 1:1 (*v*/*v*) medium. The obtained solid was recrystallized from ethanol-water 1:1 (*v*/*v*) and dried in a vacuum oven at 323 K for 8 h. Colourless single crystals, suitable for X-ray diffraction analysis, were obtained. On heating they sublimed and decomposed.

### Refinement

All H-atom positions were located in a difference Fourier map and were freely refined.

### Crystal data

**Table:**

C_4_H_10_NO^+^·C_5_H_8_NOS_2_^−^	*F*(000) = 536
*M**_r_* = 250.37	*D*_x_ = 1.379 Mg m^−^^3^
Monoclinic, *P*2_1_/*c*	Mo *K*α radiation, λ = 0.71073 Å
Hall symbol: -P 2ybc	Cell parameters from 15 reflections
*a* = 7.938 (5) Å	θ = 5.5–15.9°
*b* = 18.3232 (15) Å	µ = 0.43 mm^−^^1^
*c* = 8.8260 (5) Å	*T* = 290 K
β = 110.021 (5)°	Prism, colourless
*V* = 1206.2 (8) Å^3^	0.3 × 0.15 × 0.15 mm
*Z* = 4	

### Data collection

**Table:**

Enraf–Nonius TurboCAD-4 diffractometer	*R*_int_ = 0.041
graphite	θ_max_ = 30.0°, θ_min_ = 2.7°
non–profiled ω/2θ scans	*h* = 0→11
Absorption correction: ψ scan (North *et al.*, 1968)	*k* = 0→25
*T*_min_ = 0.795, *T*_max_ = 0.902	*l* = −12→11
3705 measured reflections	3 standard reflections every 120 min
3487 independent reflections	intensity decay: 5%
2021 reflections with *I* > 2σ(*I*)	

### Refinement

**Table:**

Refinement on *F*^2^	Primary atom site location: structure-invariant direct methods
Least-squares matrix: full	Secondary atom site location: difference Fourier map
*R*[*F*^2^ > 2σ(*F*^2^)] = 0.050	Hydrogen site location: inferred from neighbouring sites
*wR*(*F*^2^) = 0.145	All H-atom parameters refined
*S* = 1.00	*w* = 1/[σ^2^(*F*_o_^2^) + (0.0759*P*)^2^] where *P* = (*F*_o_^2^ + 2*F*_c_^2^)/3
3487 reflections	(Δ/σ)_max_ = 0.004
190 parameters	Δρ_max_ = 0.56 e Å^−^^3^
0 restraints	Δρ_min_ = −0.39 e Å^−^^3^

### Special details

**Table:**

Geometry. All e.s.d.'s (except the e.s.d. in the dihedral angle between two l.s. planes) are estimated using the full covariance matrix. The cell e.s.d.'s are taken into account individually in the estimation of e.s.d.'s in distances, angles and torsion angles; correlations between e.s.d.'s in cell parameters are only used when they are defined by crystal symmetry. An approximate (isotropic) treatment of cell e.s.d.'s is used for estimating e.s.d.'s involving l.s. planes.
Refinement. Refinement of *F*^2^ against ALL reflections. The weighted *R*-factor *wR* and goodness of fit *S* are based on *F*^2^, conventional *R*-factors *R* are based on *F*, with *F* set to zero for negative *F*^2^. The threshold expression of *F*^2^ > σ(*F*^2^) is used only for calculating *R*-factors(gt) *etc*. and is not relevant to the choice of reflections for refinement. *R*-factors based on *F*^2^ are statistically about twice as large as those based on *F*, and *R*- factors based on ALL data will be even larger.

### Fractional atomic coordinates and isotropic or equivalent isotropic displacement parameters (Å^2^)

**Table:**

	*x*	*y*	*z*	*U*_iso_*/*U*_eq_	
S1	0.20657 (9)	0.07843 (4)	0.84253 (7)	0.03827 (19)	
S2	0.34115 (10)	−0.02991 (4)	0.66044 (8)	0.0448 (2)	
O2	0.7186 (3)	0.20279 (12)	0.8050 (3)	0.0558 (5)	
O1	0.1390 (3)	0.19488 (11)	0.2852 (2)	0.0547 (6)	
N1	0.1685 (3)	0.09147 (11)	0.5323 (2)	0.0344 (5)	
N2	0.6455 (3)	0.09075 (12)	0.9933 (3)	0.0352 (5)	
C1	0.2314 (3)	0.04987 (13)	0.6648 (3)	0.0301 (5)	
C2	0.1739 (4)	0.06872 (15)	0.3746 (3)	0.0425 (6)	
C3	0.2422 (4)	0.13083 (16)	0.2981 (4)	0.0458 (7)	
C4	0.0698 (4)	0.15994 (15)	0.5227 (3)	0.0390 (6)	
C5	0.1441 (5)	0.21801 (15)	0.4412 (4)	0.0472 (7)	
C6	0.7585 (5)	0.07751 (17)	0.8933 (4)	0.0478 (7)	
C7	0.7033 (5)	0.12905 (18)	0.7531 (4)	0.0506 (7)	
C8	0.6555 (5)	0.16802 (17)	1.0443 (4)	0.0515 (7)	
C9	0.6074 (5)	0.21637 (17)	0.8988 (4)	0.0556 (8)	
H1N	0.537 (5)	0.079 (2)	0.936 (4)	0.067*	
H2N	0.681 (4)	0.063 (2)	1.085 (4)	0.067*	
H2A	0.050 (4)	0.053 (2)	0.304 (4)	0.067*	
H2B	0.248 (4)	0.027 (2)	0.394 (4)	0.067*	
H3A	0.368 (4)	0.1441 (19)	0.369 (4)	0.067*	
H3B	0.229 (4)	0.1177 (19)	0.187 (4)	0.067*	
H4A	−0.053 (5)	0.1527 (19)	0.457 (4)	0.067*	
H4B	0.088 (5)	0.1751 (18)	0.628 (4)	0.067*	
H5A	0.274 (5)	0.2265 (19)	0.512 (4)	0.067*	
H5B	0.073 (4)	0.261 (2)	0.419 (4)	0.067*	
H6A	0.887 (5)	0.0884 (19)	0.966 (4)	0.067*	
H6B	0.748 (4)	0.031 (2)	0.863 (4)	0.067*	
H7A	0.582 (5)	0.1189 (19)	0.685 (4)	0.067*	
H7B	0.786 (4)	0.125 (2)	0.697 (4)	0.067*	
H8A	0.791 (5)	0.1709 (19)	1.118 (4)	0.067*	
H8B	0.590 (5)	0.1737 (19)	1.108 (4)	0.067*	
H9A	0.481 (5)	0.2048 (19)	0.826 (4)	0.067*	
H9B	0.628 (5)	0.265 (2)	0.933 (4)	0.067*	

### Atomic displacement parameters (Å^2^)

**Table:**

	*U*^11^	*U*^22^	*U*^33^	*U*^12^	*U*^13^	*U*^23^
S1	0.0437 (4)	0.0446 (4)	0.0318 (3)	0.0029 (3)	0.0197 (3)	0.0001 (3)
S2	0.0620 (5)	0.0378 (4)	0.0438 (4)	0.0134 (3)	0.0300 (3)	0.0079 (3)
O2	0.0626 (14)	0.0449 (12)	0.0676 (13)	−0.0052 (10)	0.0324 (11)	0.0156 (10)
O1	0.0774 (15)	0.0474 (12)	0.0479 (11)	0.0132 (10)	0.0324 (10)	0.0173 (9)
N1	0.0462 (13)	0.0290 (10)	0.0307 (10)	0.0011 (8)	0.0165 (9)	0.0002 (8)
N2	0.0395 (12)	0.0350 (11)	0.0336 (10)	−0.0027 (9)	0.0157 (9)	0.0043 (8)
C1	0.0302 (11)	0.0313 (11)	0.0309 (11)	−0.0057 (9)	0.0131 (9)	−0.0006 (9)
C2	0.0670 (19)	0.0366 (14)	0.0269 (12)	−0.0020 (13)	0.0199 (12)	−0.0014 (10)
C3	0.0606 (19)	0.0446 (16)	0.0388 (14)	0.0013 (14)	0.0254 (13)	0.0046 (12)
C4	0.0445 (16)	0.0373 (14)	0.0382 (13)	0.0062 (11)	0.0180 (12)	0.0031 (11)
C5	0.0602 (19)	0.0332 (14)	0.0537 (17)	0.0073 (13)	0.0267 (14)	0.0094 (12)
C6	0.0612 (19)	0.0389 (15)	0.0571 (17)	0.0089 (14)	0.0379 (15)	0.0054 (13)
C7	0.0625 (19)	0.0549 (18)	0.0460 (16)	−0.0029 (15)	0.0336 (15)	0.0057 (13)
C8	0.071 (2)	0.0428 (16)	0.0476 (16)	0.0035 (14)	0.0285 (15)	−0.0029 (12)
C9	0.071 (2)	0.0342 (15)	0.069 (2)	0.0078 (15)	0.0329 (17)	0.0062 (14)

### Geometric parameters (Å, °)

**Table:**

S1—C1	1.728 (2)	C3—H3B	0.98 (4)
S2—C1	1.709 (2)	C4—C5	1.512 (4)
O2—C7	1.418 (4)	C4—H4A	0.96 (3)
O2—C9	1.423 (4)	C4—H4B	0.93 (3)
O1—C3	1.414 (3)	C5—H5A	1.02 (3)
O1—C5	1.428 (3)	C5—H5B	0.94 (4)
N1—C1	1.341 (3)	C6—C7	1.498 (4)
N1—C4	1.466 (3)	C6—H6A	1.02 (3)
N1—C2	1.468 (3)	C6—H6B	0.89 (4)
N2—C6	1.478 (3)	C7—H7A	0.96 (3)
N2—C8	1.480 (4)	C7—H7B	0.95 (4)
N2—H1N	0.86 (4)	C8—C9	1.498 (4)
N2—H2N	0.91 (4)	C8—H8A	1.05 (3)
C2—C3	1.515 (4)	C8—H8B	0.90 (3)
C2—H2A	1.01 (3)	C9—H9A	1.01 (3)
C2—H2B	0.94 (4)	C9—H9B	0.94 (4)
C3—H3A	1.01 (3)		
			
C7—O2—C9	110.7 (2)	H4A—C4—H4B	115 (3)
C3—O1—C5	110.1 (2)	O1—C5—C4	111.3 (2)
C1—N1—C4	124.7 (2)	O1—C5—H5A	108.8 (19)
C1—N1—C2	122.8 (2)	C4—C5—H5A	107.2 (19)
C4—N1—C2	112.2 (2)	O1—C5—H5B	103 (2)
C6—N2—C8	111.0 (2)	C4—C5—H5B	112 (2)
C6—N2—H1N	107 (2)	H5A—C5—H5B	114 (3)
C8—N2—H1N	111 (2)	N2—C6—C7	108.9 (2)
C6—N2—H2N	112 (2)	N2—C6—H6A	106.0 (19)
C8—N2—H2N	107 (2)	C7—C6—H6A	109.9 (19)
H1N—N2—H2N	109 (3)	N2—C6—H6B	109 (2)
N1—C1—S2	120.49 (17)	C7—C6—H6B	113 (2)
N1—C1—S1	119.70 (18)	H6A—C6—H6B	110 (3)
S2—C1—S1	119.79 (13)	O2—C7—C6	111.4 (3)
N1—C2—C3	109.9 (2)	O2—C7—H7A	110 (2)
N1—C2—H2A	109.1 (19)	C6—C7—H7A	110 (2)
C3—C2—H2A	111 (2)	O2—C7—H7B	104 (2)
N1—C2—H2B	106 (2)	C6—C7—H7B	109 (2)
C3—C2—H2B	113 (2)	H7A—C7—H7B	112 (3)
H2A—C2—H2B	108 (3)	N2—C8—C9	109.5 (2)
O1—C3—C2	111.9 (2)	N2—C8—H8A	100.0 (19)
O1—C3—H3A	106 (2)	C9—C8—H8A	114.1 (19)
C2—C3—H3A	109.6 (19)	N2—C8—H8B	109 (2)
O1—C3—H3B	105 (2)	C9—C8—H8B	116 (2)
C2—C3—H3B	109 (2)	H8A—C8—H8B	107 (3)
H3A—C3—H3B	115 (3)	O2—C9—C8	111.6 (3)
N1—C4—C5	110.0 (2)	O2—C9—H9A	105.6 (19)
N1—C4—H4A	109 (2)	C8—C9—H9A	109.2 (19)
C5—C4—H4A	107 (2)	O2—C9—H9B	106 (2)
N1—C4—H4B	107 (2)	C8—C9—H9B	109 (2)
C5—C4—H4B	108 (2)	H9A—C9—H9B	116 (3)
			
C4—N1—C1—S2	178.8 (2)	C2—N1—C4—C5	−53.1 (3)
C2—N1—C1—S2	5.9 (3)	C3—O1—C5—C4	−60.0 (3)
C4—N1—C1—S1	−3.0 (3)	N1—C4—C5—O1	56.4 (3)
C2—N1—C1—S1	−175.9 (2)	C8—N2—C6—C7	−55.7 (4)
C1—N1—C2—C3	−133.9 (3)	C9—O2—C7—C6	−60.0 (4)
C4—N1—C2—C3	52.4 (3)	N2—C6—C7—O2	58.1 (4)
C5—O1—C3—C2	59.7 (3)	C6—N2—C8—C9	54.9 (4)
N1—C2—C3—O1	−55.7 (3)	C7—O2—C9—C8	59.0 (4)
C1—N1—C4—C5	133.4 (3)	N2—C8—C9—O2	−56.2 (4)

### Hydrogen-bond geometry (Å, °)

**Table:**

*D*—H···*A*	*D*—H	H···*A*	*D*···*A*	*D*—H···*A*
N2—H1N···S1	0.86 (4)	2.47 (4)	3.284 (3)	158 (3)
N2—H2N···S1^i^	0.91 (4)	2.75 (4)	3.453 (2)	135 (3)
N2—H2N···S2^i^	0.91 (4)	2.39 (3)	3.221 (2)	151 (3)

Symmetry codes: (i) −*x*+1, −*y*, −*z*+2.
